# A Case of Anti-GAD 65 Autoimmune Encephalitis Associated with Focal Segmental Stiff-Person Syndrome

**DOI:** 10.3390/brainsci13020369

**Published:** 2023-02-20

**Authors:** Chen Zhang, Yuwei Dai, Binhong Han, Jian Peng, Jie Ma, Qi Tang, Li Yang

**Affiliations:** Department of Neurology, The Second Xiangya Hospital of Central South University, Central South University, No. 139 Middle Renmin Road, Changsha 410011, China

**Keywords:** glutamic acid decarboxylase, encephalitis, epilepsy, stiffness-person syndrome, recognition and treatment

## Abstract

Glutamic acid decarboxylase (GAD) antibody-related encephalitis is an autoimmune disease associated with intracellular neuronal antigens. We report on a rare case of GAD antibody-associated encephalitis complicated with focal segmental stiffness-person syndrome (SPS) in a middle-aged woman. The disease course lasted for >10 years, initially presenting with drug-resistant epilepsy, followed by stiffness of the right lower limb, and right upper limb involvement. The patient experienced anxiety and depression symptoms due to long-term illness. During hospitalization, serum and cerebrospinal fluid GAD antibodies were positive and no tumor was found. The symptoms were significantly relieved after corticosteroid therapy and intravenous immunoglobulin immunomodulation therapy. To the best of our knowledge, this case is the first to discuss the early recognition and treatment of chronic epilepsy and focal segmental SPS caused by anti-GAD antibody-related encephalitis.

## 1. Introduction

Glutamic acid decarboxylase (GAD) is a rate-limiting enzyme that produces the inhibitory neurotransmitter γ-aminobutyric acid (GABA) and is mainly located in the brain and pancreas. GAD65 and GAD67 are two subtypes of GAD that differ in molecular size, amino acid sequence, and antigenicity [1]. Compared to GAD67, GAD65 has higher antigenicity [2]. Therefore, anti-GAD antibody encephalitis generally refers to anti-GAD65 antibody encephalitis. Anti-GAD antibody-associated encephalitis is rare, with its main clinical manifestation being epilepsy, especially temporal lobe epilepsy [3]. Approximately a quarter of patients have a thymoma or small cell lung cancer, and present with paraneoplastic autoimmune encephalitis [4]. The GAD antibodies are also associated with syndromes that affect the nervous system, such as stiff-person syndrome (SPS), limbic encephalitis, and cerebellar ataxia [5,6]. Reportedly, the positive rate of anti-GAD65 antibodies can reach 80–98% in patients with SPS [7,8]. Presently, SPS is divided into classical and variant SPS [9]. The clinical manifestations of SPS vary and some patients are often misdiagnosed [10].

This report describes a rare case of GAD antibody-associated autoimmune encephalitis with focal segmental SPS. The patient initially presented with drug-resistant epilepsy, and several years later she developed stiffness of the right limb. After receiving immunotherapy, the patient achieved significant remission of seizures and right limb dysmotility.

## 2. Case Description

A 44-year-old woman was hospitalized in a local public hospital in 2011 for transient loss of consciousness after being depressed and irritable. More specifically, her family noticed brief episodes of staring and unresponsiveness. She was unable to recall the previous behavior afterwards. Following that, symptoms of transient loss of consciousness occurred at home approximately once every two weeks during mood fluctuations, manifesting as a focal impaired awareness seizure lasting for 1–2 min. Since the beginning of the disease, the seizures have been repetitive and stereotyped. Seizure symptoms are similar to initial seizure symptoms. Furthermore, she never presented with status epilepticus. She went to the local public hospital several times, but her condition did not improve. In 2014, she started to take oxcarbazepine (300 mg/q8h) for one year, and then increased it to 450 mg/q8h. Due to poor control, she added valproic acid (500 mg/q12h) for two years, and added clonazepam (0.5 mg/qd) from 2018. In 2016, the patient developed numbness and stiffness in the right lower limb. This resulted in slowness of movement and instability during walking, without muscle tremor and obvious pain. The limb stiffness gradually extended to the right upper limb. The doctors at the local hospital were unable to identify the cause of the limb stiffness. She had no neurodevelopmental abnormalities, no prenatal seizure history, no abnormalities at birth, no family history of epilepsy, and no history of drug and alcohol abuse. In 2021, the frequency of transient loss of consciousness lasting for 1–2 min has increased significantly to 1–2 times per week. She also developed cognitive symptoms such as a slow reaction time and memory loss. At this point, the patient’s clinical manifestations were deteriorating and she was admitted to our hospital for treatment. A neurological examination at our hospital showed increased muscle tension of the right lower limb and a slight increase in the muscle tension of the right upper limb, both with grade 5 muscle strength. The Babinski’s sign was present on the right side. The muscle strength and tension of the left limbs were normal, and there were no pathological signs on the left side. Fasciculations and involuntary movements were absent. Her concentration, calculation and memory were impaired. The rest of the neurological examination was normal. We suspected primary drug-resistant epilepsy because the patient had taken three antiepileptic drugs and still could not control the seizures, but we could not rule out secondary epilepsy from other disorders. The cause of limb stiffness remained unknown.

**Figure 1:**
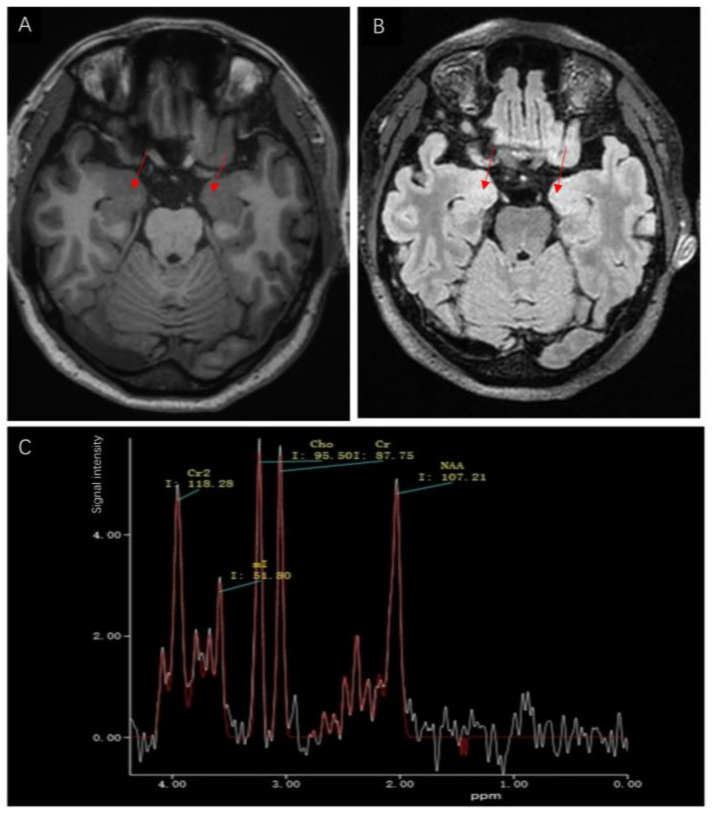
(**A**) Axial T1 weighted magnetic resonance images of the hippocampus showed no significant abnormalities in the bilateral hippocampus (red arrow). (**B**) Axial fluid-attenuated inversion recovery (FLAIR) showed no significant abnormalities in the bilateral hippocampus (red arrow). (**C**) Magnetic resonance spectroscopy image showed a decreased NAA/Cho + Cr ratio (1.22/(1.09 + 1.00) = 0.58) in the left hippocampus.

**Figure 2:**
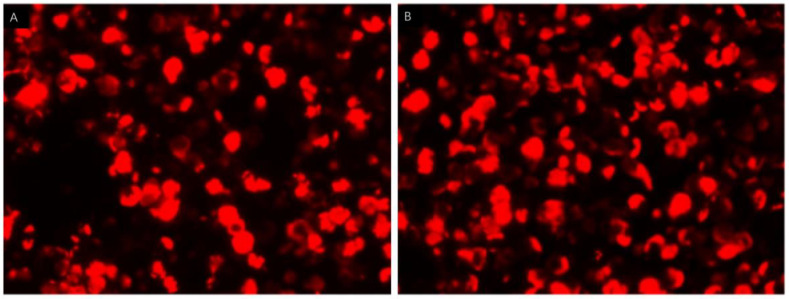
Indirect immunofluorescence assays of serum and cerebrospinal fluid (CSF). Cell-based assays of serum (**A**) and CSF (**B**) were both positive.

The etiology of the seizures and limb stiffness remains unknown. Because the Babinski sign was present on the right, cerebrovascular disease could not be excluded, so magnetic resonance imaging (MRI) was performed. A few white matter lesions were seen, and the spectrum of the bilateral temporal lobe hippocampus showed a decreased NAA/Cho + Cr ratio in the left hippocampus (Figure 1A–C). According to the Magnetic resonance spectroscopy, the neurons in the left hippocampus might be damaged or denatured. The Electroencephalogram (EEG) was abnormal with several atypical sharp-slow wave complexes of medium and high amplitudes in the bilateral frontal and temporal areas during awake and sleep, especially in the right frontal area. Temporal lobe epilepsy was considered based on the results of the EEG and magnetic resonance spectroscopy. Considering that encephalitis also causes an increase in the frequency of epilepsy, the patient underwent lumbar puncture on the second day of hospitalization. The cerebrospinal fluid (CSF) was colorless and transparent, with a pressure of 70 mm H_2_O, which is slightly lower than normal. The white blood cell count in CSF was normal. Gram staining of the CSF was negative for Gram-positive and Gram-negative bacteria and India ink staining was negative for Cryptococcus. Cerebrospinal fluid protein, glucose, and chloride levels were 289.0 mg/L, 3.63 mmol/L, and 129.8 mmol/L, respectively. No obvious abnormality was found on routine biochemical examinations of the CSF; however, the anti-GAD65 antibody was found to be positive in the antibody panel for autoimmune encephalitis using indirect immunofluorescence assay of the serum and CSF (Figure 2A,B). The anti-GAD65 antibody titers were both 1:100, and the remaining antibodies were negative. Given that the patient had repeated seizures with a loss of consciousness and poor treatment effect of antiepileptic drugs, the patient was diagnosed with autoimmune encephalitis with anti-GAD65 antibody and her epileptic symptoms were secondary to autoimmune encephalitis. Since anti-GAD 65 autoimmune encephalitis may be associated with tumors, the patient underwent computed tomography (CT) of the lungs and abdomen and a breast B-ultrasound. The tumor marker C12 was also tested. Fortunately, no tumor was found.

After finding that the anti-GAD65 antibody titers of this patient were 1:100 in both the serum and the CSF, she received corticosteroid treatment via intravenous infusion of 1000 mg methylprednisolone per day for three consecutive days, which was then reduced gradually to 240 mg for three days, 120 mg for three days, and then orally to 60 mg per day. Simultaneously, the patient was treated with drugs including oxcarbazepine (450 mg q12h), clonazepam (1 mg qd), and valproic acid (500 mg q8h). The patient had no seizures after receiving immunotherapy during hospitalization. In addition, owing to the long-term limitations of daily life, she often felt fear and occasionally had negative thoughts of being inferior to others. She consulted a psychiatrist, who prescribed sertraline hydrochloride and sodium valproate to stabilize her mood during hospitalization.

We suggested that the patient’s limb stiffness might be related to the positive GAD65 antibody. We first suspected that the patient had SPS, but the clinical manifestations were atypical; only her right limb was involved, without trunk muscle involvement. However, other tests support the diagnosis of SPS. The Electromyography (EMG) after oral administration of clonazepam showed that the relaxation of the right anterior tibial muscle was mostly poor, and the motor unit continued to release. The EMG was abnormal, and after intravenous injection of diazepam (5 mg) the right muscular tension decreased significantly. Meanwhile, the GAD65 antibody in the serum and CSF were both positive and the limb stiffness could not be explained by other neurological diseases. Therefore, a clinical diagnosis of SPS was established based on the clinical diagnostic criteria [11]. On the fourteenth day of hospitalization, the patient was administered intravenous infusion of γ-globulin, 27.5 g per day. Five days after immunoglobulin treatment, the patient was generally stable, the stiffness symptoms of the right limb disappeared, and her exercise condition significantly improved. The patient was discharged from the hospital and instructed to take prednisone acetate tablets orally to regulate immune function, and to undergo regular follow-up. Clonazepam, gabapentin, sodium valproate, and oxcarbazepine were administered to prevent seizures.

Three months after discharge, a follow-up was performed. The patient took the corticosteroids and antiepileptic drugs on time and had no drug side effects. She only had one seizure, which manifested as a dull look that lasted for more than 10 s. The seizure frequency was significantly reduced by more than 50%. The stiffness of the right limb had completely disappeared and the patient had normal motor function. Furthermore, her reaction also improved and her anxiety and depression symptoms were also significantly relieved. Unfortunately, her memory decline did not improve significantly.

## 3. Discussion

Since the discovery of anti-N-methyl-d-aspartate receptor (NMDAR) encephalitis in 2007 [12], a variety of autoantibodies against the surface or internal antigens of neuronal cells have been identified and the antibody spectrum of autoimmune encephalitis has been expanding. Unlike the acute onset of anti-NMDAR encephalitis, anti-GAD antibody-related encephalitis is mostly subacute or chronic [13]. The disease mainly manifests as seizures, movement disorders, limb or trunk stiffness, muscle spasm, cognitive decline, slurred speech, and walking instability. This study mainly discusses chronic epilepsy and SPS caused by GAD-antibody-associated encephalitis.

It is generally believed that chronic epilepsy secondary to autoimmune encephalitis is an autoimmune epilepsy [14]. In the updated epilepsy classification of 2017, the international anti-epilepsy alliance recognized that immune factors are one of the six major causes of epilepsy [15]. Autoimmune epilepsy is increasingly being considered an independent disease entity. However, there is no clear definition of the diagnostic criteria based on the course of autoimmune epilepsy and antibody titers [14]. In addition, the quality of life of patients with epilepsy is worrisome. Approximately 20% of patients with epilepsy have comorbid anxiety disorders, and 23% have depression [16]. The frequency and severity of seizures, depression, anxiety, and other complications are closely related to the quality of life [17]. In autoimmune epilepsy, early immunotherapy can reduce the frequency of seizures and improve long-term prognosis in drug-resistant patients [18,19]. Therefore, for patients with drug-resistant epilepsy, special attention should be paid to the possibility of an autoimmune etiology.

The use of specific scoring scales to screen which patients with unexplained epilepsy need to be tested for neural antibodies may be implemented in the future. Dubey et al. verified and updated the scoring scale of antibody prevalence (APE) in patients with unexplained epilepsy [20]. The scale includes 10 items, with a total of 18 points, and mainly evaluates the clinical, CSF and imaging characteristics of patients. When the APE2 score was ≥4, the sensitivity of the score to predict positive neuroantibody in patients was 98% and the specificity increased to 85%. Additionally, in 2021 de Bruijn et al. designed the antibodies contributing to the focal epilepsy signs and symptoms (ACES) score, which included six factors: cognitive symptoms, behavioral changes, autoimmune diseases, speech problems, autonomic symptoms and temporal MRI hyperintensities. Each of the six items is given one point [21]. For patients with focal epilepsy of unknown cause, an ACES score ≥ 2 indicates that the patient might have autoimmune-related causes and the negative predictive value is 100% (95% confidence interval = 81.4–100). This score is simpler than the APE2 score, and might be more convenient for clinical use. However, no detailed description of autoimmune diseases, autonomic nerve symptoms, and other items that might cause ambiguity exists. Therefore, clinicians recommend the APE2 score. The APE2 score of this patient was 3 points (seizure refractory to at least two antiseizure medications = 2, emotional lability score = 1), which does not reach the threshold score of testing neural antibodies. However, the ACES score was 2 (cognitive symptoms = 1, behavioral changes = 1), which meets the score required for further testing. This is because the two scales are applicable to different populations. In the same cohort, de Bruijn et al. also compared ACES and APE2 scores. They found that the accuracy rate of using APE2 score for detecting patients in a subacute setting with antibodies against extracellular neuronal antigens was significantly higher than that in a chronic setting. About 70% of chronic patients would have been missed by using APE2 score alone. This patient’s disease is chronic, which is the reason why her APE2 scores did not meet the requirements. Therefore, we recommend that after excluding unexplained epilepsy caused by genetic, metabolic, and other causes, that clinicians use the APE2 or ACES scale to evaluate whether patients need to check the underlying cause of neural antibodies. If the APE2 score is <4, we also need to evaluate the ACES score, especially for patients with chronic disease courses.

Currently, there is no recognized standard treatment for autoimmune epilepsy and the effectiveness of long-term immunotherapy remains unclear. First-line treatment with corticosteroids, immunoglobulins, and plasma exchange can reduce seizures [18]. For patients who fail the first-line treatment, second-line immunosuppressive treatment can also be used, such as rituximab and cyclophosphamide; however, this might mainly target neuronal surface antibodies, which is more effective [22]. The GAD antibody is an antineuronal intracellular antibody. Currently, there is no consensus as to whether anti-GAD antibodies cause disease directly or indirectly through cellular immunity. GAD65 is primarily expressed in the pre-synaptic end of GABAergic nerve terminals and can form a protein complex to anchor on the synaptic vesicle membrane [23]. When synaptic vesicles fuse with the plasma membrane, the epitopes of GAD65 can be temporarily exposed to antibodies [24]. GAD antibody is believed to have an inhibitory effect on GAD65, which changes the transmission of GABA [5]. Immunoglobulin from the CSF of GAD65 positive patients can cause motor regulation dysfunction in rats [25], which supports the direct pathogenic effect of the anti-GAD65 antibody. The GAD antibody was detected in 8.7% of patients with an unknown etiology of temporal lobe epilepsy [26], and cytotoxic T cell infiltration was found in the histopathological examination of drug-resistant patients with temporal lobe epilepsy who underwent temporal lobectomy [27]. Malter et al. measured the density of hippocampal CD3 (+) T cells in four patients with GAD antibody-associated encephalitis [28]. The number of cytotoxic T cells was higher than normal. Therefore, for patients whose symptoms cannot be controlled by first-line immunotherapy, it might be necessary to use antibodies against therapeutic interleukin-2 receptors to fight the attack of cytotoxic T cells [29]. Despite receiving immunotherapy, antiepileptic drugs are also essential. The chronic epileptic symptoms caused by anti-GAD 65 autoimmune encephalitis reported in this study were treated with antiepileptic drugs under first-line corticosteroid therapy. Within three months after discharge, the patient only had one seizure. The frequency of epileptic seizures decreased significantly.

With the exception of epilepsy, the patient developed SPS symptoms approximately five years ago. The prevalence of SPS is 1/1,250,000 [30], and its pathogenesis has not yet been fully elucidated. Since most patients with SPS have anti-GAD antibodies, it is currently believed that the possible pathological mechanism of SPS is the inhibition of GABA by anti-GAD antibodies, leading to overexcitation of motor neurons, resulting in continuous muscle rigidity [31]. This mechanism could be the cause of SPS in this patient with anti-GAD 65 autoimmune encephalitis. Certainly, not all patients with GAD65 antibody in the CSF and the serum will show SPS, which may be due to different epitopes being recognized by the GAD65 antibody. In SPS, anti-GAD antibody reactivity is predominantly directed to a conformational epitope region in the PLP- and C-terminal domains of the 65 kDa isoform [32]. In addition to anti-GAD antibodies, SPS is also related to antibodies such as anti-amphiphysin and anti-glycine receptor [33,34]. SPS can be classified into classic and variant types, including paraneoplastic SPS, progressive encephalomyelitis with rigidity and myoclonus (PERM), focal segmental SPS, spastic SPS, and SPS plus [9]. The classic SPS mainly involves the muscles of the trunk, limbs, and neck, resulting in muscle stiffness, abnormal posture, muscle pain, and spasm. Most case reports of SPS combined with other autoimmune diseases reported classic SPS involving the trunk at the same time [35,36].

However, this study reports a rare focal segmental SPS that mainly involved a single limb of the patient, with normal trunk muscles. In the past, several cases of focal segmental SPS with symptoms limited to a single limb or extending from one leg to the contralateral leg were reported [37,38,39,40]. In our study, the patient’s muscle stiffness gradually generalized from the right lower limb to the right upper limb, which differs from the previous reports. The atypical clinical manifestation may lead to a misdiagnosis, as it could be mistaken for multiple sclerosis. Multiple sclerosis is an autoimmune disease characterized by the demylenation of the central nervous system (CNS). The first symptom is often motor dysfunction in one or more limbs. Moreover, patients with multiple sclerosis are more likely to experience seizures than the general population [41,42]. Circular lesions with low T1 and high T2 signals can be seen in multiple sclerosis, which are often distributed around the ventricles, near the cortex, under the tentorium, and spinal cord [43]. Brain MRI in SPS lacks this typical feature. In SPS, electromyography shows the continuous release of motor units at rest, and benzodiazepines can improve the symptoms of muscle stiffness [11].

The prognosis of SPS is related to the initial clinical manifestations and treatment. Currently there is no unified treatment, and mainly symptomatic or autoimmune regulation is used. Studies have shown that GABA receptor agonists such as diazepam and baclofen can continuously improve muscle rigidity and spasm in patients [7,44]. Intravenous immunoglobulins can achieve long-term remission of SPS [45]. SPS is a slowly progressive disease that can lead to disabilities in severe cases. Due to the limitations of general exercise ability, the quality of life and psychological state are greatly impacted. The patient in our study had anxiety and depression due to long-term movement disorders and seizures and was unable to work independently. Therefore, early recognition and treatment of SPS is essential.

## 4. Conclusions

This report presents a patient with long-term drug-resistant epilepsy and limb stiffness who was ultimately diagnosed with GAD antibody encephalitis associated with focal segmental SPS. In a clinical setting, a lumbar puncture and GAD antibody-related detection should be investigated to avoid further delays in diagnosis and treatment.

## Data Availability

All data used in this study are available from the corresponding author on reasonable request.
